# Broad-spectrum antimicrobial activity and *in vivo* efficacy of SK1260 against bacterial pathogens

**DOI:** 10.3389/fmicb.2025.1553693

**Published:** 2025-04-10

**Authors:** Sridhar Kavela, Swetha Kakkerla, Murali Krishna Thupurani

**Affiliations:** Department of Biotechnology, Chaitanya (Deemed to be University), Hyderabad, India

**Keywords:** antimicrobial resistance, SK1260 peptide, multidrug-resistant bacteria, minimum inhibitory concentration, time-kill assay, *in vivo* infection model

## Abstract

**Introduction:**

Antimicrobial resistance (AMR) is a growing global health concern, necessitating the development of novel therapeutic agents. Antimicrobial peptides (AMPs) have emerged as promising candidates due to their broad-spectrum activity and lower resistance potential. SK1260 is a newly developed AMP evaluated for its efficacy against Gram-positive and Gram-negative pathogens, including multidrug-resistant strains.

**Methods:**

The antimicrobial activity of SK1260 was assessed through minimum inhibitory concentration (MIC) assays against clinical and reference strains of *Escherichia coli*, *Staphylococcus aureus*, *Klebsiella pneumoniae*, and *Pseudomonas aeruginosa*. Time-kill kinetics were performed to evaluate bactericidal activity over time. In vivo efficacy was determined using murine infection models, where bacterial burden, tissue pathology, and survival rates were assessed following peptide administration.

**Results:**

SK1260 exhibited potent antibacterial activity, with MIC values ranging from 3.13 to 12.5 µg/mL. Time-kill studies demonstrated dose- and time-dependent bactericidal effects, achieving complete bacterial clearance at concentrations ≥1× MIC, comparable to ciprofloxacin. In vivo studies revealed significant reductions in bacterial loads in vital organs, reduced histopathological damage, and improved survival in treated mice. Peptide treatment restored normal tissue architecture and showed efficacy equivalent to standard antibiotic therapy.

**Discussion:**

The study establishes SK1260 as a promising broad-spectrum antimicrobial agent with efficacy against drug-resistant pathogens. Its ability to reduce bacterial burden and protect tissue integrity in vivo highlights its therapeutic potential. Further preclinical development and clinical trials are warranted to explore SK1260 as a viable alternative in the fight against AMR.

## 1 Introduction

Antimicrobial resistance (AMR) is one of the most pressing challenges facing global health, threatening the efficacy of existing antibiotics and jeopardizing decades of progress in infectious disease management (Salam et al., 2023). The rise of multidrug-resistant (MDR) pathogens, including methicillin-resistant *Staphylococcus aureus* (MRSA), extended-spectrum beta-lactamase (ESBL)-producing *Escherichia coli*, carbapenem-resistant *Klebsiella pneumoniae*, and multidrug-resistant *Pseudomonas aeruginosa*, has significantly limited therapeutic options, resulting in increased morbidity, mortality, and healthcare costs (van Duin and Paterson, 2020). Compounding the issue, the pace of novel antibiotic development has lagged behind the rapid emergence of resistant strains, necessitating the urgent discovery and development of innovative antimicrobial agents with broad-spectrum activity and novel mechanisms of action (Miethke et al., 2021).

Antimicrobial peptides (AMPs) represent a promising class of therapeutic agents with unique advantages over conventional antibiotics. Unlike traditional antibiotics that target specific bacterial processes, such as protein synthesis or cell wall biosynthesis, AMPs primarily disrupt bacterial membranes, leading to rapid bactericidal effects. This mechanism makes AMPs less prone to resistance development. Additionally, many AMPs exhibit immunomodulatory properties, promote host defense mechanisms, and demonstrate activity against biofilm-associated bacteria, often recalcitrant to conventional therapies. These attributes position AMPs as valuable candidates for combating MDR pathogens and difficult-to-treat infections (Erdem Büyükkiraz and Kesmen, 2022; Zhang et al., 2021).

Among the AMPs under investigation, SK1260 (KAFAVKFAWKFHAWKAWKKAW) has emerged as a potential broad-spectrum antimicrobial agent with activity against Gram-positive and Gram-negative bacteria. Our earlier studies have suggested that SK1260 exhibits potent *in vitro* antimicrobial activity and immunomodulatory activity (Kakkerla et al., 2023). However, comprehensive data on its spectrum of activity, dose-dependent effects, and comparative performance against clinically established antibiotics remain limited. A thorough evaluation of SK1260’s antimicrobial efficacy, bactericidal kinetics, and therapeutic potential is warranted to address these gaps.

This study aims to provide a detailed characterization of SK1260’s antimicrobial properties through a combination of *in vitro* and *in vivo* assays. The minimum inhibitory concentration (MIC) of SK1260 was determined for both standard ATCC strains and clinical isolates of key bacterial pathogens, including *Escherichia coli* (*E. coli*), *Staphylococcus aureus* (*S. aureus*), *Klebsiella pneumoniae* (*K. pneumoniae*), and *Pseudomonas aeruginosa* (*P. aeruginosa*), to assess its potency. Time-kill assays were conducted to investigate the concentration- and time-dependent bactericidal activity of SK1260, shedding light on its killing kinetics and effectiveness at various doses. To evaluate its *in vivo* therapeutic potential, SK1260 was tested in murine infection models, where bacterial burden, histopathological changes, and survival outcomes were analyzed following treatment. Additionally, the performance of SK1260 was compared to ciprofloxacin, a widely used broad-spectrum antibiotic, providing a benchmark for its relative efficacy.

The *in vivo* assessments included measuring bacterial loads in key organs such as the lung, liver, kidney, and spleen, following infections with *Escherichia coli* and *Staphylococcus aureus*. Histopathological analysis of these organs was performed to determine the extent of infection-induced tissue damage and the ameliorative effects of SK1260 treatment. Furthermore, survival studies were carried out to evaluate the impact of SK1260 on infection-related mortality in mice. Collectively, these experiments offer a comprehensive evaluation of SK1260’s potential as a therapeutic agent.

The findings of this study hold significant implications for addressing the global AMR crisis. By demonstrating the efficacy of SK1260 against a broad spectrum of pathogens, including MDR strains, this research contributes to the growing body of evidence supporting AMPs as viable alternatives to conventional antibiotics. The detailed *in vitro* and *in vivo* analyses presented here aim to inform the preclinical development of SK1260 and pave the way for its eventual translation into clinical applications. Ultimately, this work underscores the importance of developing novel antimicrobial agents capable of overcoming the limitations of current therapies and meeting the urgent need for effective treatment options in the era of antimicrobial resistance.

## 2 Materials and methods

### 2.1 Peptide synthesis and purification

Peptides were synthesized by the Fmoc (N-[9-fluorenyl]-methoxycarbonyl) chemistry according to the literature procedure (Miranda and Alewood, 1999; Fields and Noble, 1990). Protected amino acids were coupled by *in situ* activation with N, N- diisopropylethylamine (DIEA), and N-hydroxy benzotriazole (HOBt). Deprotection was performed with 20% piperidine in N, N-dimethylformamide (DMF). The protected side chains of amino acid residues and the cleavage of the peptide from the solid support were performed by 95% trifluoroacetic acid (TFA)/2.5% triisopropylsilane (TIS)/2.5% water for 1 h at room temperature. After cleavage from resin, the peptides were purified by preparative reverse-phase HPLC (BioLogic Duoflow system) on a Kromasil C18 column (250 × 10 mm, particle size 5 𝜇m, pore size 100 A °). The elution was achieved with a linear gradient of 0.05% TFA in 5% methanol (A) and 0.05% TFA in 95% methanol (B) at a flow rate of 5 mL/min (57–64% B in 30 min). The main peak was pooled, lyophilized, and stored at −20∘C. The purity of the peptide was evaluated using analytical reverse-phase HPLC (Shimadzu LC-10AT) on a Lichrospher C18 column (250 × 4.6 mm) in the same mobile phase with a linear gradient at a flow rate of 1 mL/min (49–54% B in 20 min). The synthetic peptides were confirmed by electrospray mass spectrometry.

### 2.2 The strains and growth conditions

One Gram-positive bacteria and three Gram-negative bacteria were selected to measure the peptide’s antibacterial activity. The reference strains and clinical isolates were provided by the Department of Microbiology, Kakatiya Medical College, Warangal, and stored at 4°C until use. Table 1 summarizes the species, culture conditions, and details of antibiotic resistance of these bacterial strains.

**Table 1:** The species, culture conditions, and details of antibiotic resistance of these bacterial strains.

Bacterial isolate	Gram stain	Growth medium	Incubation conditions	Antibiotic resistance profile
*S. aureus ATCC 6538*	Gram-positive	Tryptic Soy Broth (TSB)	37°C	Non-resistant (control strain)
*MRSA ATCC 43300*	Gram-positive	TSB	37°C	Methicillin
*S. aureus Clinical Isolate 1*	Gram-positive	TSB	37°C	---
*S. aureus Clinical Isolate 2*	Gram-positive	TSB	37°C	Vancomycin-intermediate
*S. aureus Clinical Isolate 3*	Gram-positive	TSB	37°C	Ampicillin, Erythromycin
*E. coli ATCC 8739*	Gram-negative	Luria-Bertani (LB) Broth	37°C	Non-resistant (control strain)
*E. coli Clinical Isolate 1*	Gram-negative	LB Broth	37°C	Multidrug-resistant (MDR)
*E. coli Clinical Isolate 2*	Gram-negative	LB Broth	37°C	Carbapenem-resistant (CRE)
*K. pneumoniae ATCC 700603*	Gram-negative	LB Broth	37°C	cefotaxime, ceftazidime, and ceftriaxone
*P. aeruginosa ATCC 9027*	Gram-negative	LB Broth	37°C	Multidrug-resistant (MDR)

### 2.3 Measurement of antibacterial activity

The minimum inhibitory concentrations (MICs) of the AMPs were measured in 96-well microtiter plates according to the Clinical and Laboratory Standards Institute (CLSI) (Kowalska-Krochmal and Dudek-Wicher, 2021). Briefly, an appropriate medium for each strain containing various concentrations of AMPs (100, 50, 25, 12.5, 6.25, 3.125, 1.56, and 0.75 𝜇g/mL) is inoculated with a defined number of cells (approx. 10^5^ CFU/mL) in 96-well microtiter plates (polypropylene), whereas each plate also includes a positive growth control and a negative control (sterile control). Ciprofloxacin at a concentration range of 0.5 to 1.0 μg/mL was used as the reference antibiotic. After incubation, the MIC is determined by the lowest concentration showing no visible growth. All MICs were determined in three independent experiments performed in duplicate.

### 2.4 Time-kill kinetics assay

Time-kill kinetics of the synthetic peptide SK1260 were carried out following the procedure described by Appiah et al. (2017). Concentrations equal to 0.5 x MIC, 1x MIC, and five times the MIC of the peptide SK1260 were prepared. An inoculum size of 6 × 10^5^ CFU/mL was added and incubated at 37°C. Aliquots of 0.5 mL of the medium were taken at time intervals of 0, 1, 2, 3, 4, 5, 12, and 24 h and inoculated aseptically into 20 mL nutrient agar and incubated at 37°C for 24 h. A control test was performed for the organisms without the peptide or reference antibiotic. The colony-forming unit (CFU) of the organisms was determined. The procedure was performed in triplicate (three independent experiments) and a graph of the log CFU/mL was plotted against time.

### 2.5 Membrane uptake assay

*Escherichia coli* and *S. aureus* strains were grown in LB and MHB media, respectively, to mid-log phase and treated with SK1260 at 0.5× MIC, 1× MIC, and 5× MIC concentrations, and Ciprofloxacin at 1 μg/mL (positive control) for 60 min at 37°C. Bacterial suspensions were then stained with Propidium Iodide (PI, 10 μg/mL) for 15 min in the dark to assess membrane permeability.

### 2.6 Biofilm inhibition assay

Isolates of *S. aureus* (ATCC 43300) were grown overnight and then diluted 1:100 in tryptic soy broth (TSB) with 1% glucose. The cultures were incubated in 96-well plates at 37°C for 48 h to allow biofilm formation. After incubation, the media were removed, and the wells were rinsed with PBS to eliminate planktonic bacteria. Fresh Mueller-Hinton broth (MHB) was then added to the wells, followed by the addition of the peptide and antibiotic at the desired concentrations. The plates were further incubated at 37°C for 24 h. After incubation, the wells were washed, and the biofilms were stained with 0.5% (w/v) crystal violet for 30 min. The bound dye was then solubilized using 95% ethanol, and the optical density (OD) of the biofilms was measured.

### 2.7 Animals

Male Balb/C mice (6–8 weeks) were obtained from the Animal Facility of Jeeva Life Sciences, Hyderabad. The original breeding colonies were obtained from Jackson Labs, USA. The animals were maintained in a pathogen-free condition. All the procedures for animal experiments were approved by the Institutional Animal Ethics Committee (IAEC) and performed following the Committee for Control and Supervision of Experiments on Animals (CCSEA) guidelines.

### 2.8 *In vivo* infection and treatment

Reference strains of *Escherichia coli* and *Staphylococcus aureus* (ATCC strains) were cultured to the logarithmic growth phase in Mueller-Hinton broth (MHB) at 37°C with shaking (200 rpm). Bacterial cultures were centrifuged at 4,000 rpm for 10 min, washed twice with sterile phosphate-buffered saline (PBS), and resuspended in PBS. The bacterial suspension was adjusted to a final concentration of approximately 2.5 × 10^8^ CFU/mL. Female Balb/C mice (20–25 g, six weeks old) were infected intraperitoneally (i.p.) with 200 μL of the bacterial suspension (~2.5 × 10^8^ CFU/mouse). Six hours post-infection, treatment was initiated by administering SK1260 intraperitoneally at doses of 1 mg/kg or 2 mg/kg body weight. Treatment was repeated every 12 h. control groups received either PBS (vehicle control) or ciprofloxacin (1 mg/kg). Mice were monitored regularly for clinical signs of infection, distress, and weight loss throughout the experimental period.

### 2.9 Bacterial burden analysis

Twenty-four hours post-treatment, mice were euthanized, and lungs, liver, kidney, and spleen were aseptically collected. Tissues were homogenized in sterile PBS, serially diluted, and plated on nutrient agar. After overnight incubation at 37°C, CFUs were enumerated to assess bacterial burden in each organ.

### 2.10 Histopathological analysis

Tissue samples (lungs, liver, and spleen) were fixed in 10% neutral-buffered formalin, embedded in paraffin, and sectioned into 4 μm slices. Sections were stained with hematoxylin and eosin (H&E) and examined under a light microscope for inflammation, tissue damage, and cellular infiltration. Representative images were captured to document histopathological findings.

### 2.11 Survival assay

A separate cohort of mice was used for survival studies. Mice were infected intraperitoneally with *E. coli* or *S. aureus* and treated immediately with SK1260 (1 mg/kg or 2 mg/kg). Survival was monitored daily for seven days. The survival rates were recorded, and Kaplan–Meier analysis was performed to evaluate statistical significance.

### 2.12 Statistical analysis

All experiments were performed in triplicates, and data were expressed as mean ± standard deviation (SD). Statistical significance was determined using one-way ANOVA with Tukey’s *post hoc* test for multiple comparisons. Survival data were analyzed using Kaplan–Meier survival curves and log-rank tests. A *p*-value <0.05 was considered statistically significant.

## 3 Results

### 3.1 Minimum inhibitory concentration (MIC) assay

**Figure 1:**
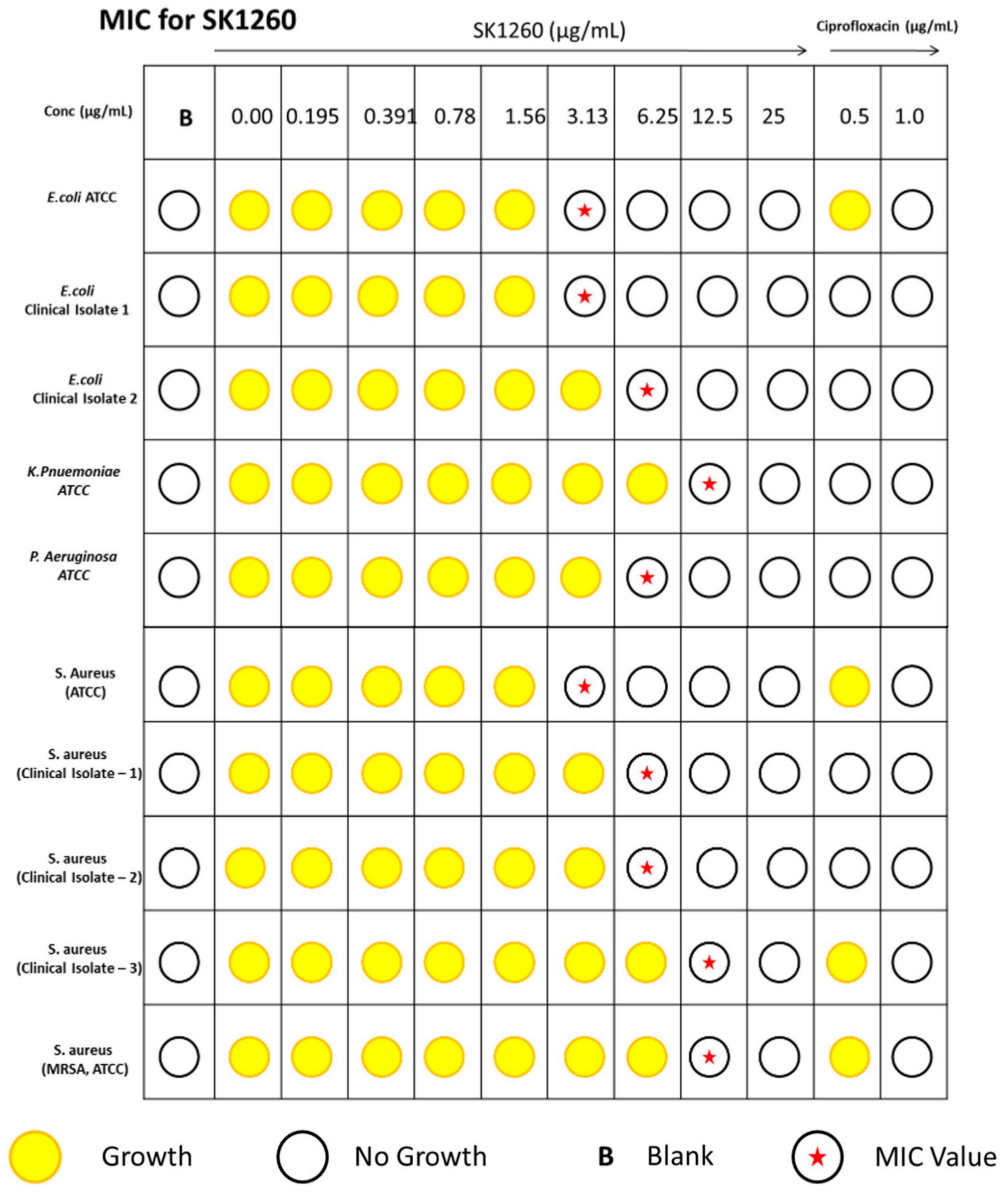
Minimum inhibitory concentration (MIC) of SK1260 against bacterial strains. MIC values of SK1260 were determined for *Escherichia coli* (ATCC and clinical strains), *Pseudomonas aeruginosa* (ATCC), *Klebsiella pneumoniae* (ATCC), *Staphylococcus aureus* (ATCC and clinical strains), methicillin-resistant *S. aureus* (MRSA, ATCC). The MIC values ranged from 3.13 to 12.5 μg/mL, indicating the broad-spectrum antimicrobial activity of SK1260.

The antimicrobial activity of SK1260 was evaluated using MIC determination against both standard (ATCC) and clinical isolates of *E. coli*, *S. aureus*, *P. aeruginosa*, and *K. pneumoniae*. The MIC values against ATCC strains were 3.13 μg/mL for *E. coli* and *S. aureus*, 6.25 μg/mL for *K. pneumoniae*, and 12.5 μg/mL for *P. aeruginosa*. For clinical isolates, the MIC values were slightly higher, with *E. coli* exhibiting MICs of 3.13 μg/mL and 6.25 μg/mL, and *S. aureus* showing MICs of 6.25 μg/mL and 12.5 μg/mL (Figure 1). These findings suggest SK1260 has potent antimicrobial activity against ATCC and clinical strains, albeit with slightly reduced potency against clinical isolates, likely due to acquired resistance mechanisms.

### 3.2 Time-kill assay

**Figure 2:**
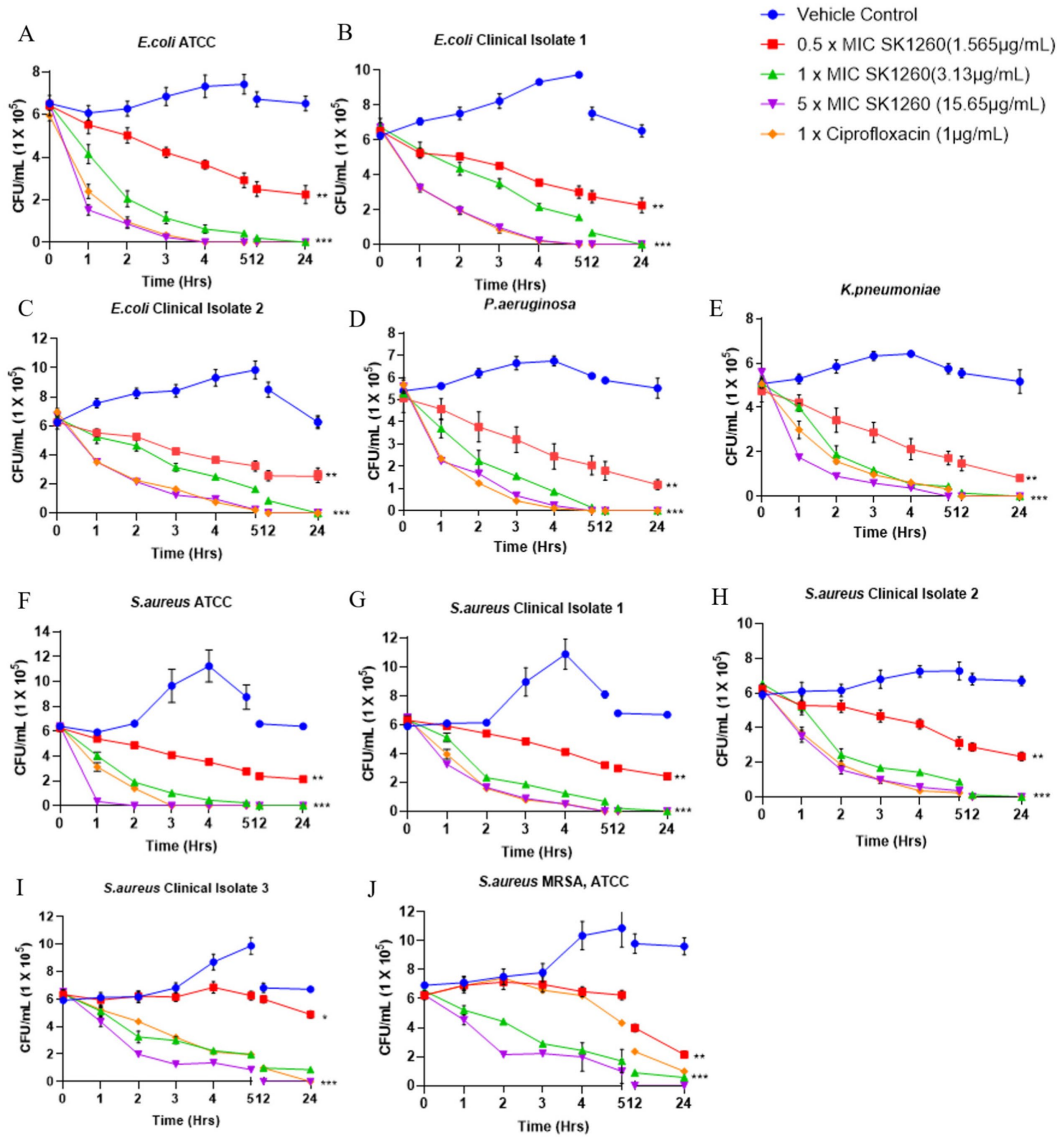
Time-kill kinetics of SK1260 against bacterial pathogens. The bactericidal activity of SK1260 was evaluated at 0.5×, 1×, and 3× MIC concentrations over time for different bacterial strains. Data are presented as the logarithmic reduction in CFUs at 0, 2, 4, 6, and 9 h. **(A–C)** Time-kill profiles of *E. coli* strains, including ATCC **(A)** and two clinical isolates **(B,C)**. **(D)** *P. aeruginosa* (ATCC strain). **(E)** *K. pneumoniae* (ATCC strain). **(F–I)** *S. aureus*, including the ATCC strain **(F)** and three clinical isolates **(G–I)**. **(J)** methicillin-resistant *S. aureus* (MRSA, ATCC strain). Data are expressed as mean ± SD (*p* value ≤0.01*, ≤ 0.001**, ≤ 0.0001***).

The bactericidal efficacy of SK1260 was evaluated in a time- and concentration-dependent manner, demonstrating strong antimicrobial activity. At 0.5× MIC, SK1260 caused a gradual reduction in CFU over 12 h but did not achieve complete bacterial clearance within 24 h, consistent with bacteriostatic effects observed at sub-inhibitory concentrations in other antimicrobial peptides (Ganz, 2003; Diamond et al., 2009). At 1× MIC, SK1260 exhibited rapid bactericidal activity, achieving complete clearance within 6 h for *S. aureus* strains, including MRSA, and within 12 h for *E. coli* and *K. pneumoniae*. At 5× MIC, complete eradication of all tested strains occurred within 4 h, highlighting its potent concentration-dependent efficacy, comparable to ciprofloxacin. *Pseudomonas aeruginosa* exhibited relative resistance at lower concentrations, showing significant CFU reductions only at 1× MIC and rapid clearance at 5× MIC within 5 h (Figures 2A–J). This resistance is consistent with known mechanisms in *P. aeruginosa*, including biofilm formation and efflux pumps, which are well-documented barriers to antimicrobial agents (Li and Nikaido, 2009; Hancock and Speert, 2000). Ciprofloxacin demonstrated faster bactericidal kinetics against *E. coli* and *P. aeruginosa*, likely due to its rapid inhibition of DNA gyrase and topoisomerase IV, which induces lethal DNA damage (Hooper, 2000). Despite this, SK1260 exhibited comparable overall efficacy at higher concentrations, achieving similar outcomes in terms of bacterial clearance. The ability of SK1260 to effectively kill multidrug-resistant strains, particularly MRSA, and to demonstrate efficacy comparable to ciprofloxacin against Gram-negative bacteria underscores its potential as a novel antimicrobial agent. These findings position SK1260 as a promising candidate for treating resistant bacterial infections, aligning with the urgent need for new therapeutics to combat antimicrobial resistance (Peyrusson et al., 2020; Zasloff, 2019).

### 3.3 Membrane uptake using propidium iodide

**Figure 3:**
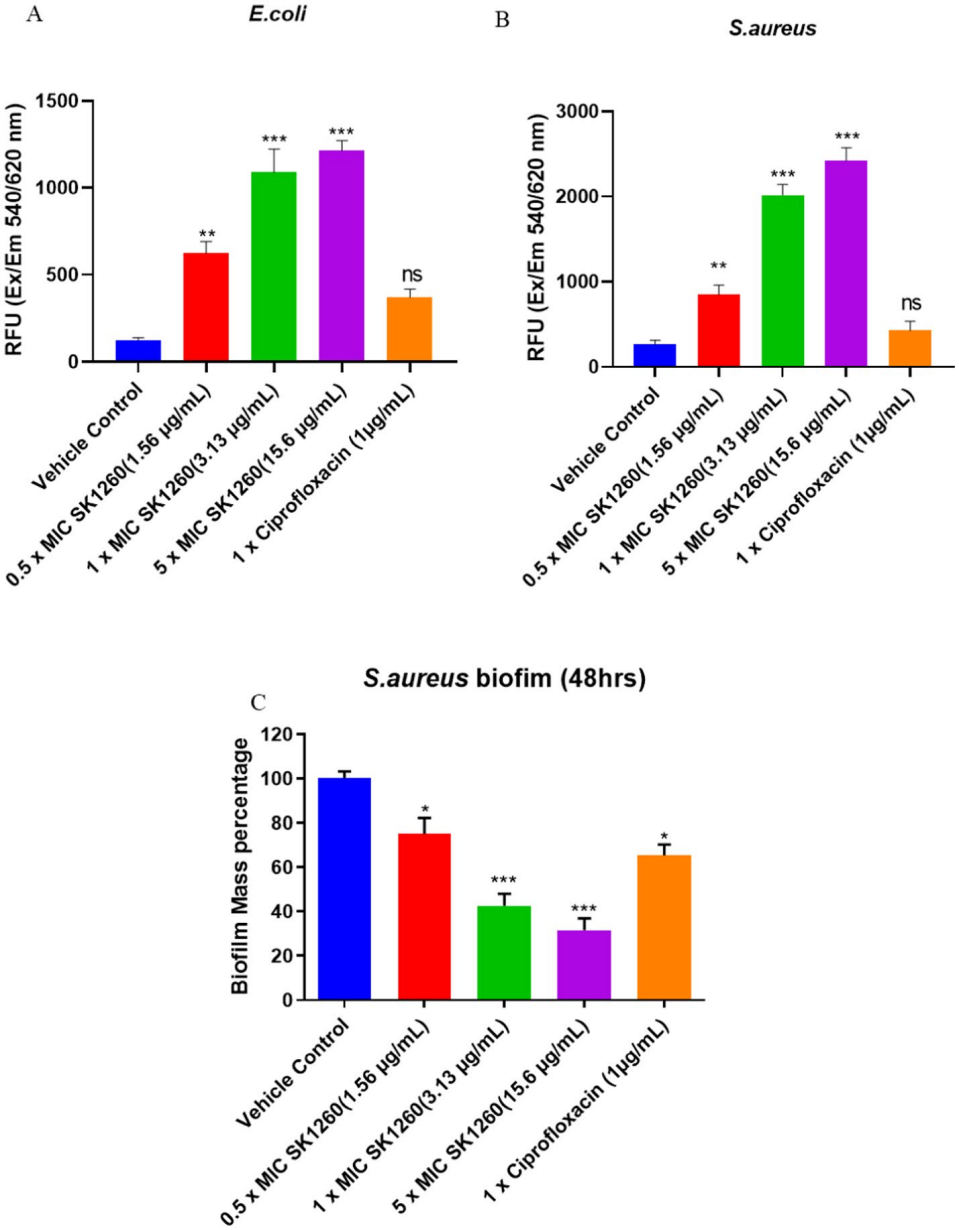
Membrane disruption and biofilm inhibition by SK1260 and ciprofloxacin. Membrane disruption was assessed by propidium iodide (PI) staining in *E. coli* **(A)** and *S. aureus* **(B)** after treatment with SK1260 and ciprofloxacin. SK1260 caused a dose-dependent increase in PI fluorescence, indicating membrane permeability, with higher concentrations showing greater disruption compared to ciprofloxacin. Biofilm inhibition was evaluated in *S. aureus* after 48 h of biofilm formation **(C)**. SK1260 at 1× MIC reduced biofilm mass by 50%, while ciprofloxacin reduced it by 40%. Data are presented as the mean ± SD of three independent experiments (*p* value ≤0.01*, ≤ 0.001**, ≤ 0.0001***).

Fluorescence measurements indicated a dose-dependent increase in membrane permeability upon treatment with SK1260 and Ciprofloxacin in *E. coli* and *S. aureus*. SK1260 at higher concentrations led to a substantial rise in PI fluorescence, suggesting significant membrane disruption. In contrast, lower concentrations induced only a moderate increase in fluorescence, indicating partial membrane compromise. Ciprofloxacin also showed a moderate membrane permeability, though its effect was comparable to SK1260 at 1× MIC and lower than the highest SK1260 concentration (Figures 3A,B). The overall trend suggests that SK1260 exhibits strong membrane-disruptive activity, with its impact intensifying in a dose-dependent manner, surpassing Ciprofloxacin at higher concentrations. Differences in membrane disruption between the two treatments may be attributed to their distinct mechanisms of action, with SK1260 primarily targeting membrane integrity and Ciprofloxacin acting through DNA gyrase inhibition with possible secondary membrane effects.

### 3.4 Biofilm inhibition

Biofilm formation is a significant virulence factor of *S. aureus* (Peng et al., 2022). The polysaccharide matrix of biofilm protects microorganisms from host immune defenses and impedes the effectiveness of antimicrobials, preventing them from targeting bacteria residing deep within the biofilm (Sharma et al., 2023). Additionally, biofilms serve as a persistent niche for bacteria, leading to the continuous release of microbes within the host, which causes chronic infections, relapses, systemic infections, and treatment failure (Vestby et al., 2020). Given the challenges posed by staphylococcal biofilms and their role in promoting recurrent infections, we evaluated whether our peptide could disrupt mature biofilms of *S. aureus*, formed after 48 h. As shown in Figure 3C, SK1260 (at 1 × MIC) significantly disrupted 48-h-old biofilms, reducing the biofilm mass by half. In comparison, 1X Ciprofloxacin reduced only 40% of the biomass (*p* < 0.01).

### 3.5 Bacterial bioburden in mice

**Figure 4:**
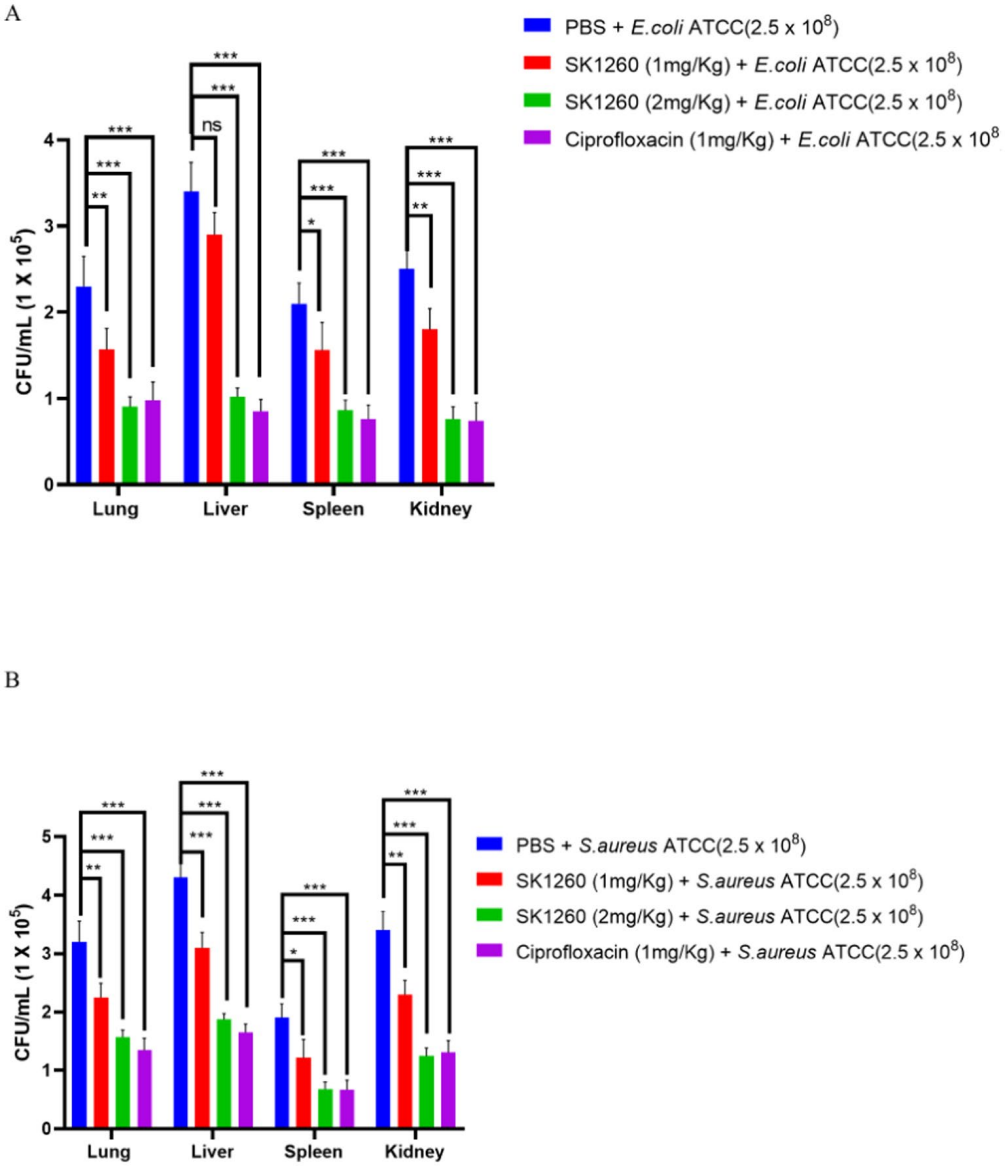
Bacterial burden reduction in infected mice treated with SK1260. Bacterial CFUs were quantified from lung, liver, and spleen homogenates of mice infected with *E. coli* **(A)** or *S. aureus* **(B)** and treated with SK1260 (1 mg/kg or 2 mg/kg), ciprofloxacin, or PBS. SK1260 significantly reduced bacterial counts in all tested organs, with 2 mg/kg demonstrating comparable efficacy to ciprofloxacin. Data are expressed as mean ± SD (*p* value ≤0.01*, ≤ 0.001**, ≤ 0.0001***).

The lung, liver, spleen, and kidney bacterial load were evaluated in mice infected with *E. coli* and *S. aureus* ATCC strains. PBS-treated controls exhibited high bacterial burdens in all organs, indicating severe systemic infection. SK1260 at 1 mg/kg significantly reduced bacterial load in all organs, with the greatest reductions observed in the kidney and liver, consistent with their roles in filtering and drug accumulation (Roger, 2024). At 2 mg/kg, SK1260 achieved near-complete clearance in the kidney and liver and complete clearance in the lung and spleen (Figures 4A,B). For *E. coli*, SK1260 reduced liver bacterial loads from 3.4 ± 0.34 log CFU/organ in controls to 1.02 ± 0.10 CFU/organ. Similarly, *S. aureus* burdens in the spleen were eradicated, and liver burdens were reduced to 1.87 ± 0.10 CFU/organ. Ciprofloxacin demonstrated similar reductions at 1 mg/kg, achieving near-complete or complete clearance across all organs, consistent with its established pharmacodynamic profile (Drusano, 2007).

### 3.6 Histopathology analysis

**Figure 5:**
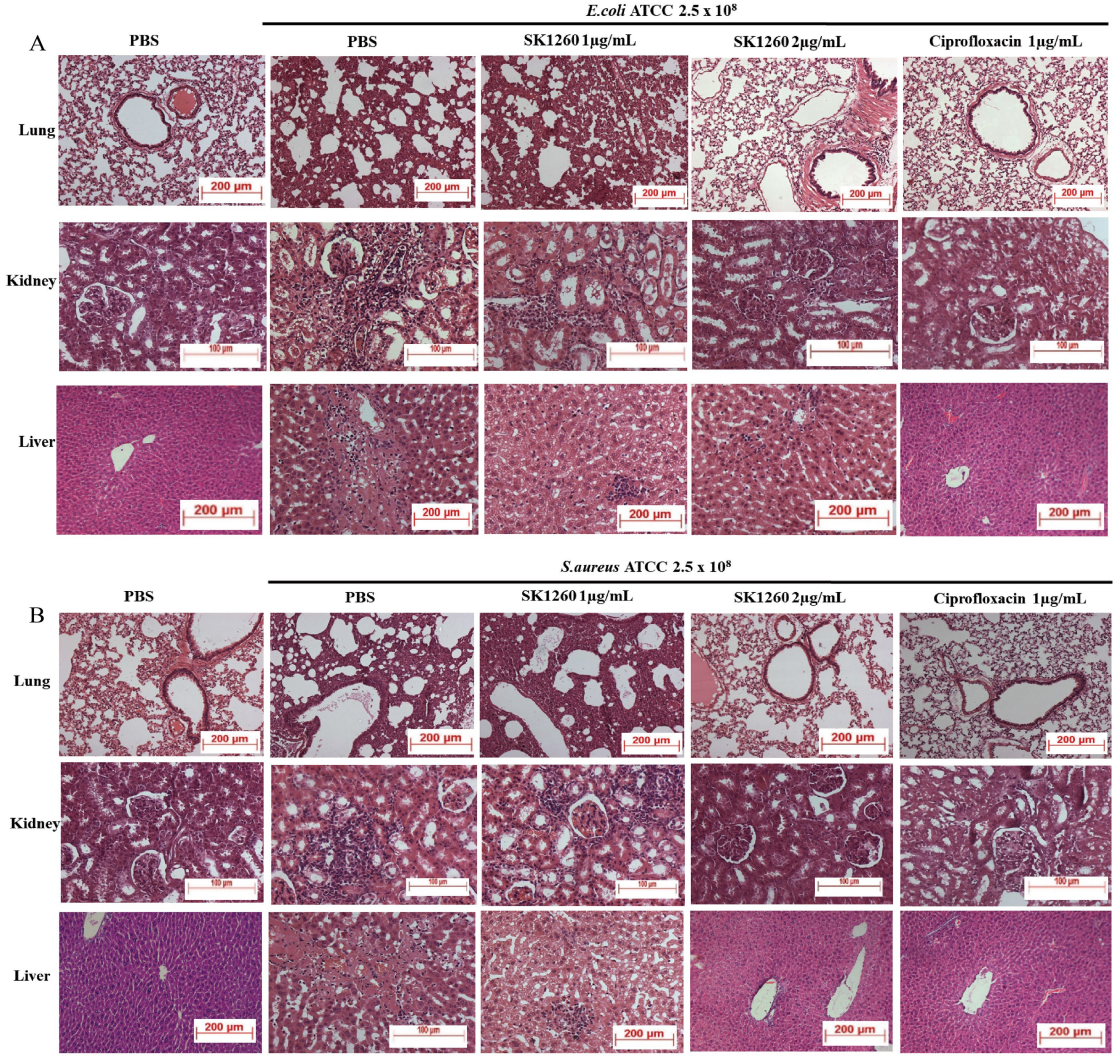
Histopathological analysis of infected organs after SK1260 treatment. Representative hematoxylin and eosin (H&E)-stained sections of lung, kidney, and liver tissues from mice infected with *E. coli* **(A)** or *S. aureus* **(B)**, treated with SK1260, ciprofloxacin, or PBS. Untreated (PBS) tissues showed severe inflammation, tissue damage, and cellular infiltration, while SK1260 treatment restored normal tissue architecture, particularly at the 2 mg/kg dose.

Histopathological analysis of infected tissues revealed significant inflammation and damage in PBS-treated controls, indicative of severe infection. In *E. coli*-infected mice, lung tissues displayed alveolar and interstitial inflammation, kidney sections showed marked tubular and interstitial inflammation, and the liver exhibited necrosis with lymphocyte infiltration, findings consistent with systemic bacterial infection (Riedemann et al., 2003). SK1260 treatment at 1 mg/kg substantially reduced these pathological changes, while at 2 mg/kg, tissue architecture was restored to near-normal levels (Figure 5A). In *S. aureus*-infected mice, severe inflammation and necrosis were observed in the PBS control group, reflecting the pathogen’s ability to induce substantial tissue injury through virulence factors and host immune activation (Lowy, 1998). SK1260 at 1 mg/kg reduced inflammation and tissue damage, and at 2 mg/kg, normal tissue structure was comparable to uninfected controls (Figure 5B). Ciprofloxacin demonstrated similar restorative effects, underscoring its established efficacy in mitigating infection-induced damage (Drusano, 2007).

### 3.7 Survival analysis

**Figure 6:**
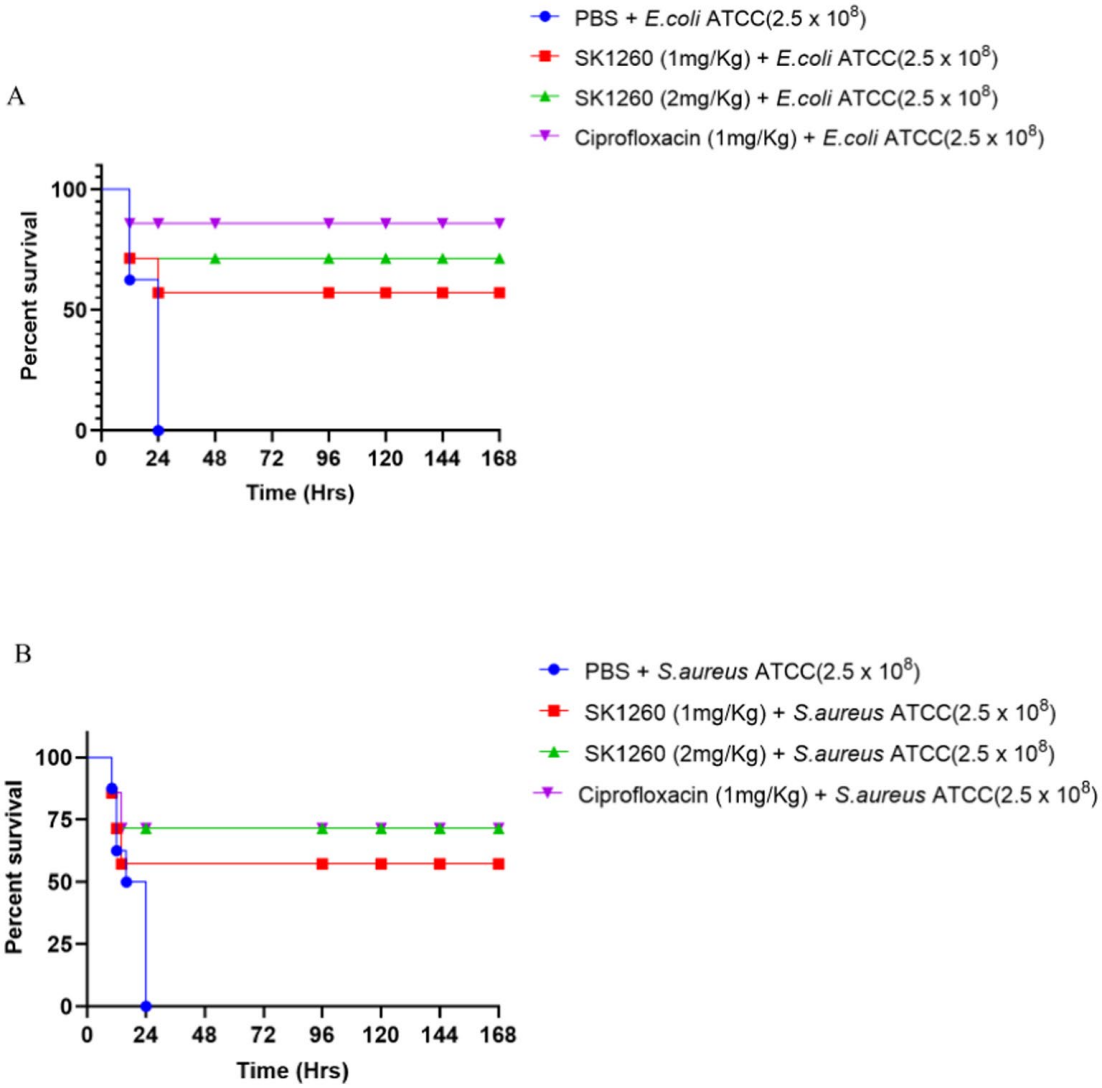
Survival analysis of infected mice treated with SK1260. Kaplan–Meier survival curves of mice infected with *E. coli* **(A)** or *S. aureus* **(B)** and treated with SK1260 (1 mg/kg or 2 mg/kg), ciprofloxacin, or PBS. SK1260-treated mice exhibited significantly improved survival rates in a dose-dependent manner, with 75% survival at the 2 mg/kg dose over 7 days (*p* < 0.01).

The survival study highlighted the severity of systemic infections, with 0% survival in PBS-treated controls over seven days. SK1260 treatment significantly improved survival in a dose-dependent manner. In *E. coli*-infected mice, survival rates reached 57.14% at 1 mg/kg and 71.4% at 2 mg/kg, compared to 85.7% with ciprofloxacin (Figure 6A). Similarly, in *S. aureus*-infected mice, SK1260 achieved 57.14% survival at 1 mg/kg and 71.4% at 2 mg/kg, with ciprofloxacin showing 71.4% survival (Figure 6B). These results demonstrate SK1260’s efficacy in reducing bacterial burden and enhancing survival, comparable to ciprofloxacin, and underscore its potential as a therapeutic alternative against multidrug-resistant infections.

## 4 Discussion

The findings of this study highlight SK1260 as a potent antimicrobial agent with significant efficacy in both *in vitro* and *in vivo* settings against Gram-positive and Gram-negative bacteria, including multidrug-resistant strains. MIC assays demonstrated that SK1260 exhibited low MIC values against standard ATCC strains and clinical isolates, indicating strong antimicrobial activity. While clinical isolates showed slightly higher MIC values compared to ATCC strains, this is likely due to resistance mechanisms such as efflux pumps or enzymatic modifications, commonly observed in clinical pathogens. Importantly, SK1260 retained activity against these resistant strains, aligning with studies of novel antimicrobial agents that show resilience to such mechanisms (Zasloff, 2019; Blair et al., 2015).

Time-kill assays revealed that SK1260 displays a concentration- and time-dependent bactericidal effect. At sub-MIC levels, a gradual decline in bacterial counts was observed, whereas concentrations at or above MIC led to rapid bacterial clearance. The eradication of bacterial strains within six hours at higher concentrations is comparable to ciprofloxacin, a well-established antibiotic. However, *P. aeruginosa* showed relative resistance at lower concentrations, consistent with its known resistance mechanisms, including biofilm formation and efflux pumps (Hancock and Speert, 2000). Despite this, SK1260 achieved complete clearance at higher concentrations, highlighting its robust antimicrobial potential and broad-spectrum activity.

Membrane permeability assays, conducted using propidium iodide (PI), demonstrated a dose-dependent increase in fluorescence, indicating that SK1260 significantly compromises membrane integrity. At higher concentrations, SK1260 caused more pronounced membrane disruption, surpassing ciprofloxacin in its effectiveness at equivalent concentrations. This suggests that SK1260 primarily targets the bacterial membrane, a mechanism distinct from ciprofloxacin’s action on DNA gyrase. The stronger membrane-disruptive effect observed with SK1260, particularly at higher concentrations, may contribute to its rapid bactericidal activity and its ability to overcome bacterial resistance mechanisms that target intracellular processes.

In addition to its membrane-disruptive properties, SK1260 was found to effectively disrupt mature biofilms of *S. aureus*, a key factor in chronic and recurrent infections. At 1× MIC, SK1260 reduced biofilm mass by 50%, a significantly higher reduction compared to ciprofloxacin (40%). This suggests that SK1260 has a superior capacity to target and degrade biofilm structures, which are typically resistant to conventional antibiotics. The ability of SK1260 to inhibit biofilm formation and disrupt established biofilms highlights its potential for treating infections caused by biofilm-forming pathogens, including multidrug-resistant strains.

In murine infection models, SK1260 significantly reduced bacterial burdens in major organs such as the lung, liver, kidney, and spleen. Substantial reductions were observed at a dose of 1 mg/kg, while a dose of 2 mg/kg resulted in near-complete bacterial clearance, demonstrating effective systemic distribution and tissue penetration. These results are comparable to ciprofloxacin, a key agent in bacterial infection treatment, and indicate that SK1260 may serve as a viable alternative in clinical settings (Drlica et al., 2008). The dose-dependent reductions in bacterial loads underscore its therapeutic potential and favorable safety profile.

Histopathological evaluations revealed significant tissue damage in untreated control groups, characterized by inflammation, necrosis, and fibrosis in organs such as the lung, liver, and kidney. Treatment with SK1260 at 1 mg/kg ameliorated these pathological changes, while treatment at 2 mg/kg restored normal tissue architecture. This dual ability to reduce bacterial burden and mitigate infection-induced tissue damage underscores SK1260’s therapeutic potential. Such properties are critical for severe systemic infections where tissue preservation is essential, and the observed effects are comparable to those of ciprofloxacin and other antimicrobial peptides like LL-37 (Kahlenberg and Kaplan, 2013).

Survival analysis further confirmed SK1260’s efficacy, significantly improving survival rates in infected mice. While the PBS-treated control group exhibited 0% survival, treatment with SK1260 at 1 mg/kg resulted in a survival rate of 57.14%, which increased to 71.4% at 2 mg/kg. Although ciprofloxacin achieved slightly higher survival rates, SK1260’s performance is notable, particularly against multidrug-resistant pathogens such as MRSA. The peptide’s ability to control systemic infection and enhance survival rates highlights its potential as a next-generation antimicrobial agent.

While SK1260 exhibited slightly slower bactericidal activity against some Gram-negative strains than ciprofloxacin, its overall efficacy, including significant reductions in bacterial burdens, restoration of normal tissue morphology, and improved survival rates, is highly encouraging. Given the rising global concern of ciprofloxacin resistance (Davies and Davies, 2010), the development of new agents like SK1260 is critical. Its broad-spectrum activity, ability to combat multidrug-resistant strains, and tissue-protective effects make it a strong candidate for further research and clinical development.

Future studies should focus on elucidating SK1260’s mechanism of action to understand its interactions with bacterial targets better. Evaluating its pharmacokinetics and long-term safety in larger animal models is essential for clinical translation. Investigating its efficacy in combination therapies with existing antibiotics could further enhance its applicability, particularly against biofilm-associated infections (Peyrusson et al., 2020). Moreover, its activity in eradicating biofilms warrants attention, as biofilm-associated infections pose significant treatment challenges. Next, we will focus on optimizing the structural stability and antimicrobial potency of SK1260 through amino acid modifications and exploring strategies like peptide conjugation with targeting agents. Additionally, we will evaluate the impact of these modifications on enhancing membrane disruption, biofilm inhibition, and resistance to enzymatic degradation. Overall, the promising results of this study position SK1260 as a potential solution to the global antimicrobial resistance crisis and a valuable addition to the therapeutic arsenal.

## 5 Conclusion

The findings of this study provide compelling evidence for the potential of SK1260 as an effective antimicrobial agent against multidrug-resistant strains of *Escherichia coli*, *Staphylococcus aureus*, *Pseudomonas aeruginosa*, and *Klebsiella pneumoniae*. The MIC assays demonstrated potent bactericidal activity, and the time-kill studies revealed a dose- and time-dependent bactericidal effect, membrane disruption, and effective biofilm inhibition that was comparable to ciprofloxacin. In murine models, SK1260 significantly reduced bacterial burden in key organs, alleviated tissue damage, and improved survival rates in a dose-dependent manner, with the 2 mg/kg dose providing results similar to the positive control, ciprofloxacin. Histopathological analysis further corroborated these findings, showing restoration of normal tissue architecture upon treatment. These results underscore the broad-spectrum efficacy of SK1260 against both Gram-positive and Gram-negative pathogens, including those with multidrug resistance. SK1260 holds promise as a novel therapeutic candidate for the treatment of bacterial infections, particularly in the context of the growing challenge posed by antimicrobial resistance. Further preclinical and clinical studies are warranted to evaluate its safety profile and therapeutic potential.

## Data Availability

The original contributions presented in the study are included in the article/supplementary material, further inquiries can be directed to the corresponding author.
